# The aging trajectories of brain functional hierarchy and its impact on cognition across the adult lifespan

**DOI:** 10.3389/fnagi.2024.1331574

**Published:** 2024-01-19

**Authors:** Xiao Wang, Chu-Chung Huang, Shih-Jen Tsai, Ching-Po Lin, Qing Cai

**Affiliations:** ^1^Shanghai Key Laboratory of Brain Functional Genomics (Ministry of Education), Institute of Brain and Education Innovation, School of Psychology and Cognitive Science, East China Normal University, Shanghai, China; ^2^Shanghai Center for Brain Science and Brain-Inspired Technology, Shanghai, China; ^3^NYU-ECNU Institute of Brain and Cognitive Science, New York University Shanghai, Shanghai, China; ^4^Brain Research Center, National Yang-Ming Chiao-Tung University, Taipei, Taiwan; ^5^Department of Psychiatry, Taipei Veterans General Hospital, Taipei, Taiwan; ^6^Division of Psychiatry, School of Medicine, National Yang-Ming Chiao-Tung University, Taipei, Taiwan; ^7^Institute of Neuroscience, National Yang-Ming Chiao-Tung University, Taipei, Taiwan; ^8^Department of Education and Research, Taipei City Hospital, Taipei, Taiwan

**Keywords:** aging process, adult lifespan, functional hierarchy, connectome gradient, cognition decline, gender effect

## Abstract

**Introduction:**

The hierarchical network architecture of the human brain, pivotal to cognition and behavior, can be explored via gradient analysis using restingstate functional MRI data. Although it has been employed to understand brain development and disorders, the impact of aging on this hierarchical architecture and its link to cognitive decline remains elusive.

**Methods:**

This study utilized resting-state functional MRI data from 350 healthy adults (aged 20–85) to investigate the functional hierarchical network using connectome gradient analysis with a cross-age sliding window approach. Gradient-related metrics were estimated and correlated with age to evaluate trajectory of gradient changes across lifespan.

**Results:**

The principal gradient (unimodal-to-transmodal) demonstrated a significant non-linear relationship with age, whereas the secondary gradient (visual-to-somatomotor) showed a simple linear decreasing pattern. Among the principal gradient, significant age-related changes were observed in the somatomotor, dorsal attention, limbic and default mode networks. The changes in the gradient scores of both the somatomotor and frontal–parietal networks were associated with greater working memory and visuospatial ability. Gender differences were found in global gradient metrics and gradient scores of somatomotor and default mode networks in the principal gradient, with no interaction with age effect.

**Discussion:**

Our study delves into the aging trajectories of functional connectome gradient and its cognitive impact across the adult lifespan, providing insights for future research into the biological underpinnings of brain function and pathological models of atypical aging processes.

## 1 Introduction

The human brain undergoes complex processes of development, maintenance and reorganization throughout the lifespan, which are essential for human cognition and behavior. Cognition relies on the hierarchical organization of brain network connectome for the integration of information across spatial and temporal scales (Mesulam, 1998; Mesulam, 2012). For example, information in visual perception-related circuits is processed at lower levels of the hierarchy, whereas higher-level cognitive tasks rely on communication and information sharing between local and distal brain regions at the higher level of the hierarchy. However, such hierarchical organization may deteriorate during brain aging, accompanied by cognitive decline due to disruptions in the efficiency of information processing and integration between networks (Park and Reuter-Lorenz, 2009; Betzel et al., 2014). Therefore, identifying the changes in the hierarchical structures of the brain network throughout lifespan not only reveals the neural basis underlying brain maintenance during aging (Rajah and D'Esposito, 2005), but may also expedite early screening for accelerated aging-related neurodegenerative diseases (Abellaneda-Perez et al., 2019).

To explore changes in human brain network across the lifespan, researchers have extensively studied the brain’s topological structure in-depth using approaches such as resting-state functional connectivity (FC) or graph theory. These approaches highlighted changes in higher-level hierarchy of the brain network, i.e., the maturation of default mode network (DMN) during adolescence (Fan et al., 2021) and the reconfiguration of whole-brain FC pattern in old age (Betzel et al., 2014). From early to late adulthood, the key organizational features of the brain’s functional connectome include decreased connectivity within networks and increased connectivity between networks (Chan et al., 2014; Spreng et al., 2016), which reflects network dedifferentiation during aging process (Goh, 2011). Based on graph-theoretical analyses, studies suggest that normal aging is characterized by a decrease in segregation and modularity, and an increase in the participation coefficient of functional brain networks (Chan et al., 2014; Geerligs et al., 2015). Moreover, the decreased segregation of association networks with age has been related to the decline of episodic memory ability (Chan et al., 2014) and processing speed performance (Ng et al., 2016). These findings imply that brain network dedifferentiation, possibly coupled with altered functional hierarchies between lower- and higher-level areas, is a central aspect of aging. This dedifferentiation may be associated with the cognitive performance in older individuals.

However, brain connectome studies based on connectivity or graph theory have not fully captured the transitions of network hierarchy at the macroscale dimension during aging process. Recent research, using a non-linear dimension decomposition approach, has demonstrated a principal gradient of connectivity differentiation along the cortical surface in adult individuals, delineating the brain’s hierarchical architecture (Margulies et al., 2016). This connectome gradient places primary sensory and motor networks on one end and the higher transmodal networks on the other, which reflects the variances of connectivity patterns among them, in line with the earlier cortical core hierarchy hypothesis (Mesulam, 1998). The connectome gradient has been used to capture the cognitive performance spectrum from perception and action to abstract cognitive abilities (Margulies et al., 2016; Hong et al., 2019; Paquola et al., 2019; Xia Y. et al., 2022) and the contracted principal gradient was associated with higher cognitive load in young adults (Zhang et al., 2022). Setton et al. (2022) observed that, although functional gradients in older adults remained stable, they identified dedifferentiation in transmodal regions, yet found no significant link to cognitive decline. It is evident that the hierarchical changes in the brain’s connectome across lifespan remain elusive. The variation in network gradient properties might be nonlinear, necessitating further exploration into the changes in functional network gradients from adulthood to old age. In adulthood, Bethlehem et al. found that the dispersion distance within transmodal communities increased with age, and this escalating pattern was linked to the fluid intelligence (Bethlehem et al., 2020). However, it still needs to be elucidated how and whether the brain’s hierarchical organization changes at global and local scales across the adult lifespan, and furthermore, whether the changes in functional hierarchy can help individuals support various domain of cognition.

To deepen our understanding of the aging effect on the human brain network organization, we employed resting-state functional MRI and connectome gradient analysis. This study aims to investigate how the brain’s functional hierarchy changes with age in healthy adults and how these changes impact cognitive aging. We hypothesized that: (1) The principal connectome gradient may undergo pronounced age-related changes but still maintain its dominant position throughout adulthood; (2) On local scales, the relative hierarchical positioning of transmodal subnetworks might shift across the adult lifespan. Additionally, we investigated the correlations between functional connectome gradient with both the general cognition and working memory ability. We postulated that the alterations in hierarchical structure of subnetworks might be associated with domain-specific cognitive decline.

## 2 Materials and methods

### 2.1 Participants

This study initially included 387 healthy adults from the Taiwan Aging and Mental Illness Neuroimaging Database (Huang et al., 2020). Thirty-seven participants with personal or family history of psychiatric disorders, neurological disease, medium or severe cognitive impairment (Mini-mental State Examination, MMSE <24, or Clinical Dementia Rating scale >0.5), incomplete demographic information and unqualified T1-weighted or resting-state functional magnetic resonance imaging (rs-fMRI) data were excluded. The data of 350 participants (age = 44.74 ± 15.91 years, female = 213, education level = 15.66 ± 3.73 years) were finally included in the statistical analysis. The study obtained ethical approval from the Institutional Review Board of Taipei Veterans General Hospital and all participants gave the written informed consent. Details are described in Supplementary materials.

### 2.2 Imaging data acquisition

MRI scans were performed at National Yang-Ming University in Taiwan using a 3 T Siemens MAGNETOM Tim Trio MRI Scanner (Siemens Healthcare, Erlangen, Germany) with a 12-channel head coil. Participants were instructed to relax with their eyes closed, without falling asleep. High-resolution structural T1-weighted (T1w) images were acquired with three-dimensional magnetization prepared rapid gradient-echo sequence (3D-MPRAGE; repetition time, TR = 2,530 ms, echo time, TE = 3.5 ms, TI = 1,100 ms, field of view, FoV = 256 mm, flip angle = 7°, 192 sagittal slices, voxel size = 1.0 mm^3^, no gap). Resting-state functional MRI was acquired using a gradient echo-planar imaging (EPI) sequence (TR = 2,500 ms, TE = 27 ms, FoV = 220 mm, flip angle = 77°, matrix size = 64 × 64 × 43, voxel size = 3.44 mm × 3.44 mm × 3.40 mm). The rs-fMRI scan consisted of 200 contiguous EPI volume, which was acquired along the anterior commissure–posterior commissure plane. Total scan time for T1w and rs-fMRI was 17 min for each participant (Localizer: <1 min, T1w: 8 min, Resting: 8 min).

### 2.3 Imaging data preprocessing

fMRI images preprocessing was performed using fMRIPrep 20.2.1 (Esteban et al., 2019). The overall preprocessing included the following steps: removal of the first 10 volumes from the whole series; slice-timing correction; head motion correction; co-registration to the T1w images; physiological noise regressors extraction; estimation of several confounding parameters and time-series; spatial smoothing with a Gaussian-smoothing kernel of 6 mm full-width at half-maximum (FWHM); and regressing out the confounding variables. Participants with brain structural abnormalities, mean frame-wise displacement (FD) parameters >0.3 mm, or maximum head motion >1.5 mm or 1.5 degrees, and or more than 10% of the frames FD > 0.5 mm were excluded. Details are described in Supplementary materials.

### 2.4 Connectome gradient analysis

We constructed a region-wise FC matrix for each participant, based on Schaefer’s cortical parcellation map of 400 regions (Schaefer et al., 2017), by computing the Pearson correlation coefficients (converted to Fisher’s *Z*-values) between the averaged time series of each pair of brain regions. The functional connectome gradients (Margulies et al., 2016) were estimated using the BrainSpace toolbox^1^ (Vos de Wael et al., 2020). The FC profile vector of each brain region was thresholded by retaining the top 10% strongest connections and the remaining connections were set to zero, as was done previously (Margulies et al., 2016). We then calculated the cosine similarity matrix that captures similarity in connectivity patterns between each pair of regions. The diffusion embedding mapping algorithm with a manifold learning parameter of *α* = 0.5 (Margulies et al., 2016; Hong et al., 2019) was then applied to the similarity matrix to identify multiple low-dimensional gradient components. To ensure the comparability of the gradient pattern among participants, we used the Procrustes rotation alignment approach to align each individual’s original gradient distribution pattern to a group-level gradient template that based on the overall healthy adults. For each connectome gradient, a gradient score was assigned to each brain region, which represents the relative hierarchical position along the gradient axes.

To quantify the global connectome gradient pattern, we calculated the global gradient metrics (post-alignment), including gradient explanation ratio, range and variation for all participants. The gradient explanation ratio, defined as the eigenvalue of the given gradient divided by the sum of all eigenvalues, represents the percentage of connectivity variance accounted for by that gradient. The gradient range indicates the difference of gradient scores in the encoded connectivity pattern between the regions localized at the gradient ends. The gradient variation, i.e., the variance of the given gradient, reflects the heterogeneity in the connectivity structure across regions.

### 2.5 Statistical analysis

#### 2.5.1 Cross-age sliding window analysis

**Figure 1:**
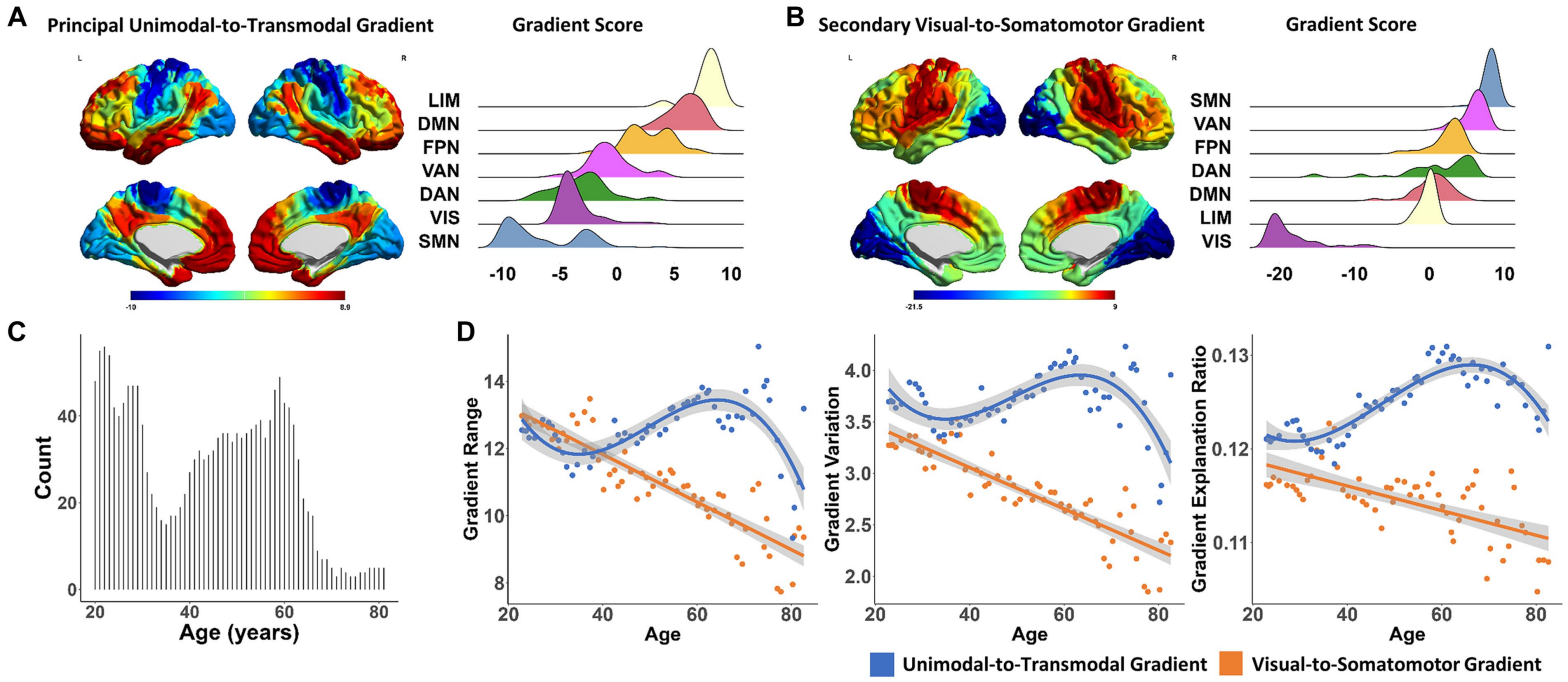
The aging process of functional connectome gradients across the adult lifespan. **(A)** The principal unimodal-to-transmodal gradient pattern (left). The gradient score distributions based on functional subnetworks (Yeo’s 7 networks atlas) in the principal gradient (right). **(B)** The secondary visual-to-somatomotor gradient pattern (left). The gradient score distributions based on functional subnetworks in the secondary gradient (right). **(C)** The histogram of sample size distribution in the cross-age sliding window analysis. **(D)** The global metrics of the principal gradient, including gradient range (left), variation (middle) and explanation ratio (right), showed significant non-linear cubic association with age (blue dots and lines). The global metrics of the secondary gradient showed a simple linear decreasing aging pattern (orange dots and lines). Surface rendering was generated using BrainNet Viewer (www.nitrc.org/projects/bnv/). LIM, limbic network; DMN, default mode network; FPN, frontal–parietal network; VAN, ventral attention network; DAN, dorsal attention network; VIS, visual network; SMN, somatomotor network.

To delineate the aging process of connectome gradients at the population level aged 20 to 85, we conducted a cross-age sliding window analysis. Specifically, we defined the age windows based on participants’ age in ascending order, with each window spanning 5 years and taking a step size of 1 years. This processing generated 62 subgroups, with the average age of participants in each window ranging from 22.85 to 82.6 years (Figure 1C). For each age window, we averaged the aligned gradients across all participants and computed three global gradient metrics, including the gradient explanation ratio, range and variation.

#### 2.5.2 The aging trajectories of the connectome gradient patterns

We used generalized linear regression model (GLM) to quantify the age-related changes of three global gradient metrics. At the population level, considering the potential linear and nonlinear effects of age, we used the linear, quadratic, cubic and bi-quadratic models, with global gradient metrics as dependent variable, age as independent variable, and education level and mean FD parameters as covariates. These models were defined as follows:

Linear model:


$$Y=b+\beta_{1}age+\beta_{2}education+\beta_{3}meanFD+\varepsilon$$

Quadratic model:


$$Y=b+\beta_{1}age+\beta_{2}age^{2}+\beta_{3}education+\beta_{4}meanFD+\varepsilon$$

Cubic model:


$$Y=b+\beta_{1}age+\beta_{2}age^{2}+\beta_{3}age^{3}+\beta_{4}education+\beta_{5}meanFD+\varepsilon$$

Bi-quadratic model:


$$\begin{array}{l} Y=b+\beta_{1}age+\beta_{2}age^{2}+\beta_{3}age^{3}+\beta_{4}age^{4}\\ \;\;\;\;\;\;\;\;\;\;\;\;\;\;+\beta_{5}education+\beta_{6}meanFD+\varepsilon \end{array}$$

Where Y is the global gradient metrics, b is the random effect, and ε is the residual.

We used the Akaike Information Criterion (AIC) index, Bayesian Information Criterion (BIC) index, R-squared and root mean square error (RMSE) to determine the best-fit model that best captured the age effects. Models with higher R-squared and lower AIC, BIC and RMSE are considered to achieve better balance between the goodness of fit and simplicity of the model. To determine which model performs best-fit to the current dataset, we conducted 1,000 times bootstrap resampling in each age window to assess the above best-fit metrics on linear, quadratic, cubic and bi-quadratic models, and identified the final fitting model using ANOVA to compare these criterions between models. Finally, we pinpointed the turning point age by identifying the inflection point with the lowest slope of the model curve.

Additionally, we examined the relationship between age and gradient scores of functional subnetworks (Yeo’s 7 networks) (Thomas Yeo et al., 2011) and regions using the same models mentioned above, with the false discovery rate (FDR, Genovese et al., 2002) correction *q* < 0.05. At the individual level, the aging trajectories of global gradient metrics and gradient scores of functional subnetworks and regions were also examined, with the FDR correction *q* < 0.05.

#### 2.5.3 Association between the connectome gradients and functional network topographical properties

To determine whether the connectome gradients capture the functional network property, we associated the topographical integration and segregation of the connectome network with the global gradient metrics, controlling the effect of age, gender, education level and mean FD parameters. These topographical properties included clustering coefficient, characteristic path length, global efficiency and small-worldness. To maintain consistency between the network density used in this analysis and used in the connectome gradient analysis, the top 10% strongest connections were retained. The network topographical properties were calculated based on the thresholded binary sparse matrix using Brain Connectivity Toolbox (BCT).^2^

To assess the sensitivity of connectome gradients to healthy aging compared to the network topology, we conducted a commonality analysis and estimated the unique and common variance between gradient metrics and topographical properties that explains normal aging. The model as follows:


$$\begin{array}{l} Age=b+\beta_{1}gradient+\beta_{2}graph+\beta_{3}gender\\ \;\;\;\;\;\;\;\;\;\;\;\;\;\;\;\;\;\;\;\;+\beta_{4}education+\beta_{5}meanFD+\varepsilon \end{array}$$

Where b is the random effect, and ε is the residual.

#### 2.5.4 The cognitive implications of the connectome gradient patterns

We measured the working memory ability of each participant through digit span forward (DSF) and backward (DSB) tests and assessed the general cognition using MMSE. The MMSE consists of 11 questions divided into six subscales, namely orientation, registration, attention and calculation, recall, language, and visuospatial skills (Folstein et al., 1975; Cameron et al., 2013; Arevalo-Rodriguez et al., 2021). The relationship between the connectome gradients and cognition abilities were examined in this study. To conduct the regression analysis in the population level, we computed the mean scores of DSF, DSB and MMSE subscales of 62 cross-age sliding windows. The averaged DSF, DSB and MMSE subscale scores in age windows were associated with global gradient metrics and gradient scores of functional subnetworks using linear regression models, controlling the effect of age, education level and mean FD parameters. The statistical significance was set at *p* < 0.05 after Bonferroni correction for the multiple comparisons [Working memory: global metrics, *p* < 0.05/12 (2 gradients × 3 metrics × 2 digital span tests); subnetworks: *p* < 0.05/28 (2 gradients × 7 subnetworks × 2 digital span tests). MMSE subscales: global metrics*, p* < 0.05/36 (2 gradients × 3 metrics × 6 subscales); subnetworks, *p* < 0.05/84 (2 gradients × 7 subnetworks × 6 subscales)]. At the individual level, the association between the connectome gradients and the cognition abilities were also examined using linear regression models and Bonferroni correction (*p* < 0.05), with age, gender, education level and mean FD parameters as covariates.

#### 2.5.5 Gender effects on the connectome gradient patterns

To examine the gender effects on global gradient metrics and gradient scores of functional subnetworks and regions at the individual level, we conducted the multivariate analysis of covariance (MANCOVAN^3^), with age, education level and mean FD parameters as covariates. To identify gender differences in the aging process of the connectome gradients, we tested the age-by-gender interaction effects on the connectome gradient.

#### 2.5.6 The potential influences of the cross-age sliding window parameters

To ascertain the robustness of the main results and investigate the potential influences of the cross-age sliding window parameters, we conducted validation analysis on the effects of sample size in age windows, gender, window width and step size on the main findings. The statistical analysis details are described in Supplemental materials.

## 3 Results

In the studied population, the principal unimodal-to-transmodal gradient explained 12.48 ± 1.07% of the total connectivity variance. As shown in Figure 1A, this gradient was organized along a gradual axis, from the primary visual/somatomotor networks (VIS/SMN) to the DMN and limbic network (LIM). The second most significant gradient accounted for 11.53 ± 1.32% of the connectome variance, with a gradual axis defined by the VIS at one end and the SMN at the other (Figure 1B).

### 3.1 The aging process of the functional connectome gradients

At the population level, the global metrics of the principal unimodal-to-transmodal gradient, including gradient explanation ratio (*t* = −5.446, *p* = 1.187 × 10^−6^), range (*t* = −4.906, *p* = 8.383 × 10^−6^), and variation (*t* = −4.113, *p* = 1.292 × 10^−4^), showed significant non-linear cubic associations with age (Table 1 and Figure 1D; Results for bootstrap resampling 1,000 times, Supplementary Table S1). The principal gradient range and variation initially decreased with age, with a minimum value at age 34.58, and then increased until a dramatic decline after age 62.05. The corresponding minimum and maximum value of gradient explanation ratio at age 30.62 and 68.44. Additionally, three subgroups of young, middle-aged and elderly individuals were grouped based on the age of min- and max-value of explanation ratio. The principal gradient’s explanation ratio was significantly greater than that of the secondary gradient in the young (Pair *t-*test. Population level, *t* = 14.866, *p* = 1.219 × 10^−7^; individual level, *t* = 4.099, *p* = 8.507 × 10^−5^), middle-aged (Population level, *t* = 10.85, *p* = 4.742 × 10^−13^; individual level, *t* = 9.033, *p* = 1.11 × 10^−16^) and elderly group (Population level, *t* = 15.12, *p* = 1.252 × 10^−9^; individual level, *t* = 3.863, *p* = 0.0014).

**Table 1:** Goodness-of-fit metrics for generalized linear regression model.

Gradient metrics	Fitting model	Unimodal-to-transmodal gradient				Visual-to-somatomotor gradient			
		*R* ^2^	RMSE	AIC	BIC	*R* ^2^	RMSE	AIC	BIC
Range	Linear	0.274	0.817	154.774	163.283	0.822	0.597	115.838	124.346
	Quadratic	0.37	0.762	146.958	157.594	0.82	0.602	117.703	128.338
	Cubic	0.551	0.643	126.792	139.555	0.817	0.606	119.573	132.336
	Bi-quadratic	0.588	0.648	128.753	143.644	0.814	0.611	121.334	136.224
Variation	Linear	0.249	0.263	14.283	22.791	0.8	0.181	−32.35	−23.842
	Quadratic	0.352	0.245	6.096	16.732	0.798	0.182	−30.758	−20.122
	Cubic	0.493	0.216	−8.27	4.493	0.795	0.183	−29.032	−16.269
	Bi-quadratic	0.511	0.212	−9.597	5.294	0.792	0.185	−27.032	−12.142
Explanation ratio	Linear	0.45	0.0026	−560.015	−551.506	0.529	0.0025	−565.369	−556.86
	Quadratic	0.582	0.0022	−576.039	−565.403	0.548	0.0024	−566.939	−556.303
	Cubic	0.722	0.0018	−600.391	−587.629	0.54	0.0024	−564.985	−552.222
	Bi-quadratic	0.764	0.0017	−609.84	−594.95	0.537	0.0024	−563.68	−548.79

RMSE, root mean squared error; AIC, Akaike information criterion; BIC, Bayesian information criterions.

The secondary visual-to-somatomotor gradient metrics showed simple significant linear decreasing aging patterns (gradient explanation ratio: *t* = 0.153, *p* = 0.879; range: *t* = −6.961, *p* = 3.41 × 10^−9^; variation: *t* = −6.203, *p* = 6.3 × 10^−8^, Figure 1D).

**Figure 2:**
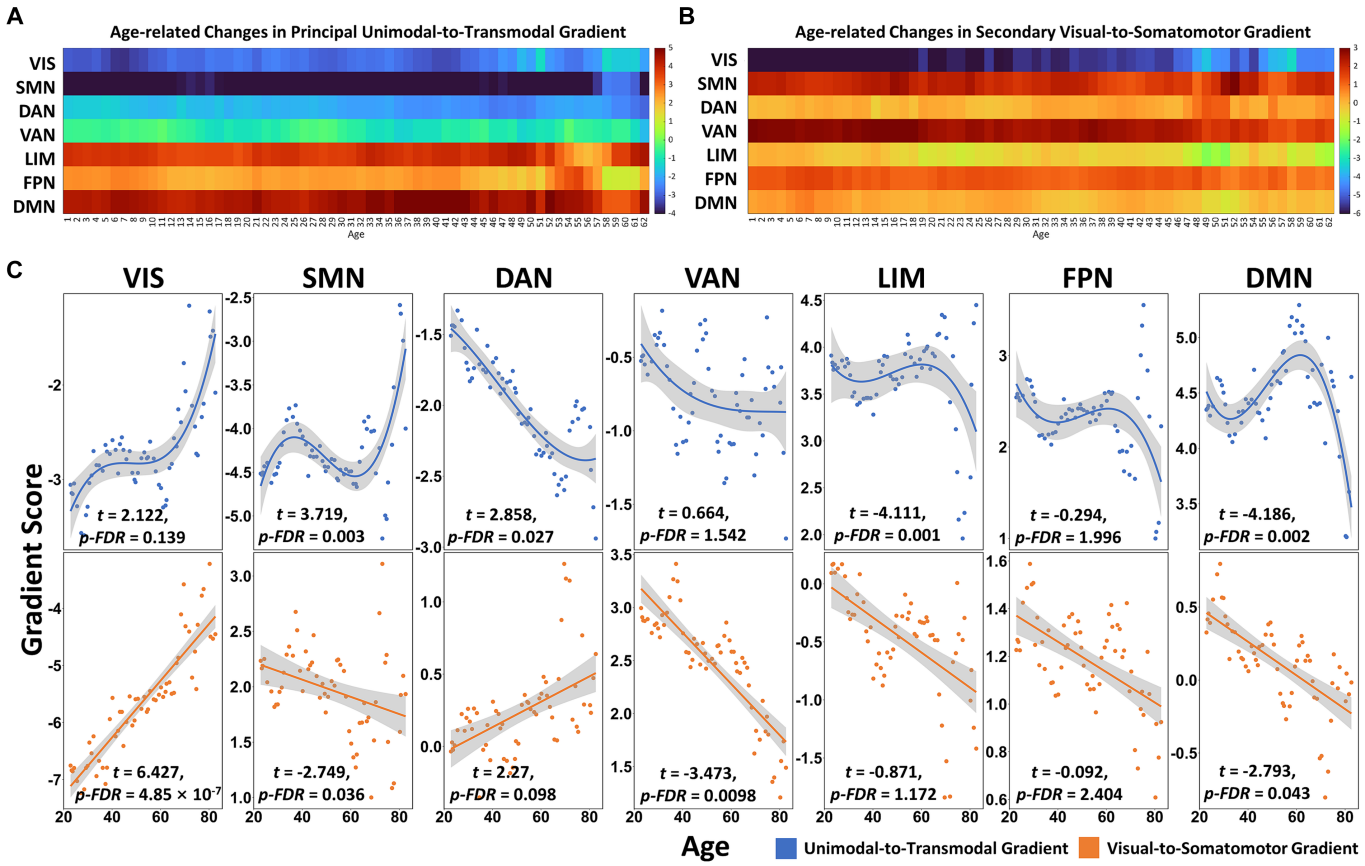
The aging process of connectome gradients in functional subnetworks across the adult lifespan. **(A)** The principal gradient scores of functional networks in age windows. **(B)** The secondary gradient scores of functional networks in age windows. **(C)** The cubic and linear aging trajectories of the principal (blue dots and lines) and secondary (orange dots and lines) gradient scores in functional networks (Yeo’s 7 networks). FDR corrected *q* < 0.05. VIS, visual network; SMN, somatomotor network; DAN, dorsal attention network; VAN, ventral attention network; LIM, limbic network; FPN, frontal–parietal network; DMN, default mode network.

**Figure 3:**
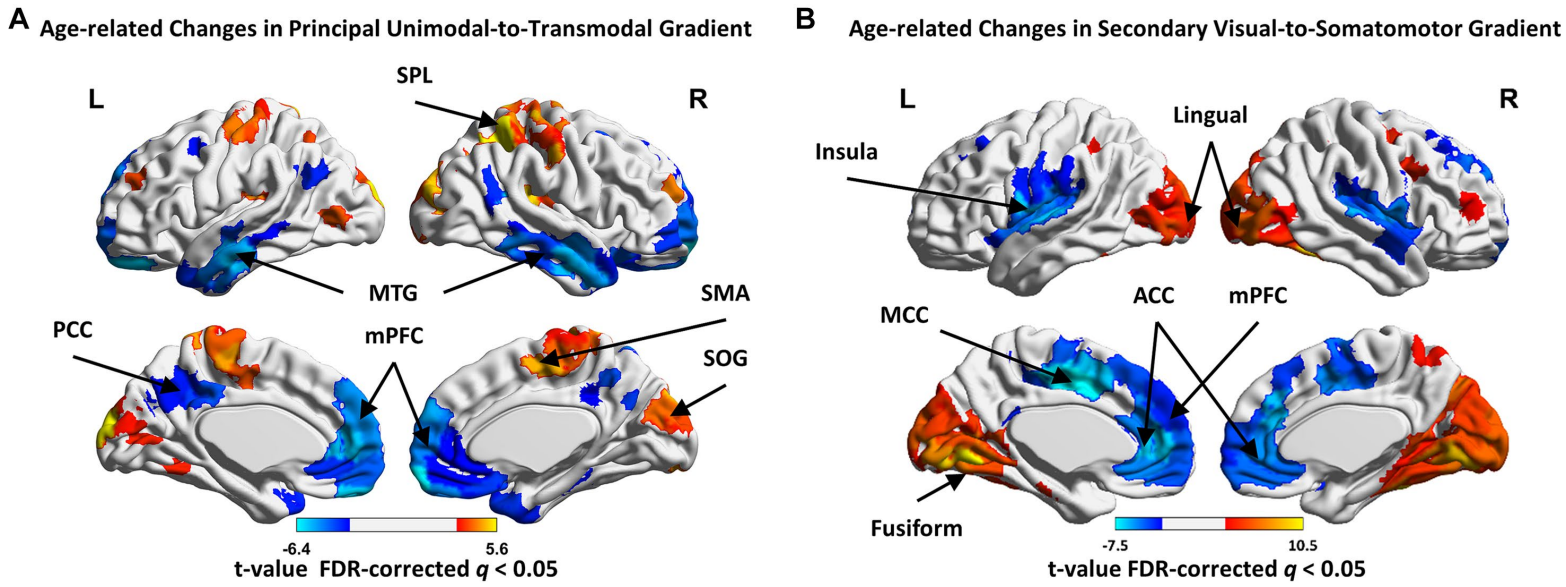
The age-related changes in regional gradient scores in principal and secondary gradients. **(A)** The regions showed age-related increase in principal gradient score, including the supplemental motor area (SMA), pre- and postcentral gyrus, superior (SPL) and inferior parietal gyrus (IPL), superior (SOG) and inferior occipital gyrus (IOG); the regions showed age-related decrease, including the medial prefrontal cortex (mPFC), anterior (ACC) and posterior cingulate cortex (PCC), middle temporal gyrus (MTG) and superior frontal gyrus (SFG). **(B)** The regions showed age-related increase in secondary gradient score, including the fusiform gyrus, lingual gyrus, SOG, middle occipital gyrus (MOG) and cuneus; the regions showed age-related decrease, including the postcentral gyrus, superior temporal gyrus (STG), insula, medial superior frontal gyrus (mSFG), medial orbital frontal gyrus (mOFG), ACC and middle cingulate cortex (MCC). Higher/lower *t*-value are presented as warm/cold colors. FDR-corrected *q* < 0.05.

In the principal gradient, significant non-linear cubic aging trajectories were detected for SMN (*t* = 3.719, *p*_FDR = 0.003), dorsal attention network (DAN, *t* = 2.858, *p*_FDR = 0.027), LIM (*t* = −4.111, *p*_FDR = 0.001) and DMN (*t* = −4.186, *p*_FDR = 0.002) gradient scores (Figures 2A,C). The secondary gradient scores of VIS (*t* = 6.427, *p*_FDR = 4.85 × 10^−7^), SMN (*t* = −2.749, *p*_FDR = 0.036), ventral attention network (VAN, *t* = −3.473, *p*_FDR = 0.0098) and DMN (*t* = −2.793, *p*_FDR = 0.043) showed significant linear association with age (Figures 2B,C). At the region level, the regions showed age-related increase in principal gradient scores were mainly located around the supplemental motor area (SMA), pre- and postcentral gyrus, superior (SPL) and inferior parietal gyrus (IPL), superior (SOG) and inferior occipital gyrus (IOG), while age-related decreasing gradient score were mainly found in the medial prefrontal cortex (mPFC), anterior (ACC) and posterior cingulate cortex (PCC), middle temporal gyrus (MTG) and superior frontal gyrus (SFG, FDR corrected *q* < 0.05. Figure 3A and Supplementary Table S2). In the secondary gradient, the regions showed age-related increasing score were mainly covered the fusiform gyrus, lingual gyrus, SOG, middle occipital gyrus (MOG) and cuneus, while age-related decreasing gradient score were mainly located around the postcentral gyrus, superior temporal gyrus (STG), insula, medial superior frontal gyrus (mSFG), medial orbital frontal gyrus (mOFG), ACC and middle cingulate cortex (MCC, FDR corrected *q* < 0.05, Figure 3B and Supplementary Table S2). The age-related changes of the principal and secondary connectome gradients at the individual level were shown in Supplementary Results and Supplementary Figures S1, S2.

### 3.2 Association between the connectome gradients and functional network topographical properties

We examined the association between topographical properties and global gradient metrics to determine if connectome gradients capture functional network properties. The results showed that the global metrics of principal and secondary gradients were significantly associated with clustering coefficient, characteristic path length, global efficiency and small-worldness property (Table 2 and Supplementary Figure S3). No significant correlation was found between the secondary gradient metrics and small-worldness property. The commonality analysis showed that the unique contribution of global gradient metrics was greater than topographical properties or the common contribution of these two on the normal aging (Supplementary Table S3).

**Table 2:** The association between the connectome gradient global metrics (gradient range, variation and explanation ratio) and functional network topographical properties (clustering coefficient, characteristic path length, global efficiency and small-worldness).

	Clustering coefficient		Characteristic path length		Global efficiency		Small-worldness	
	*t*	*p*	*t*	*p*	*t*	*p*	*t*	*p*
Principal unimodal-to-transmodal gradient								
Range	17.298	<0.001	19.758	<0.001	−19.689	<0.001	−3.265	0.0012
Variation	14.795	<0.001	18.257	<0.001	−18.152	<0.001	−2.976	0.0031
Explanation ratio	4.888	<0.001	8.204	<0.001	−8.13	<0.001	−2.858	0.0045
Secondary visual-to-somatomotor gradient								
Range	15.915	<0.001	12.576	<0.001	−12.682	<0.001	−1.366	0.173
Variation	15.042	<0.001	12.369	<0.001	−12.458	<0.001	−1.504	0.134
Explanation ratio	8.074	<0.001	7.66	<0.001	−7.684	<0.001	−0.317	0.751

### 3.3 The cognitive implications of the connectome gradient patterns

**Figure 4:**
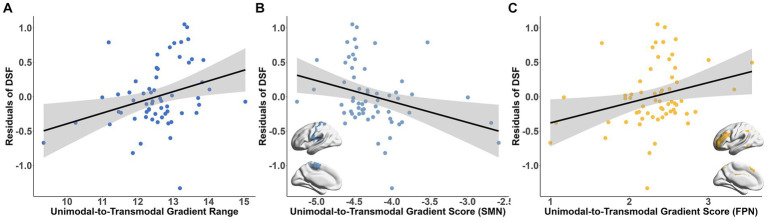
Association between the connectome gradient and working memory ability. **(A)** The association between the principal unimodal-to-transmodal gradient range and the DSF score residuals. **(B,C)** The association between the gradient scores of SMN **(B)**, FPN **(C)** and the DSF score residuals. Bonferroni correction *p* < 0.05. DSF, digital span forward; SMN, somatomotor network; FPN, frontal–parietal network.

To investigated the cognitive implications of connectome gradients at the population level, we examined the association between the global gradient metrics and the scores of DSF, DSB and MMSE subscales. The results showed a positive association between the principal gradient range and the DSF score (*t* = 3.208, Bonferroni-corrected *p* = 0.026; Figure 4A). Further analysis revealed that the gradient scores of SMN and frontal–parietal network (FPN) were significantly related with the DSF score (SMN: *t* = −3.861, Bonferroni-corrected *p* = 0.004; FPN: *t* = 4.008, Bonferroni-corrected *p* = 0.0025; Figures 4B,C).

**Figure 5:**
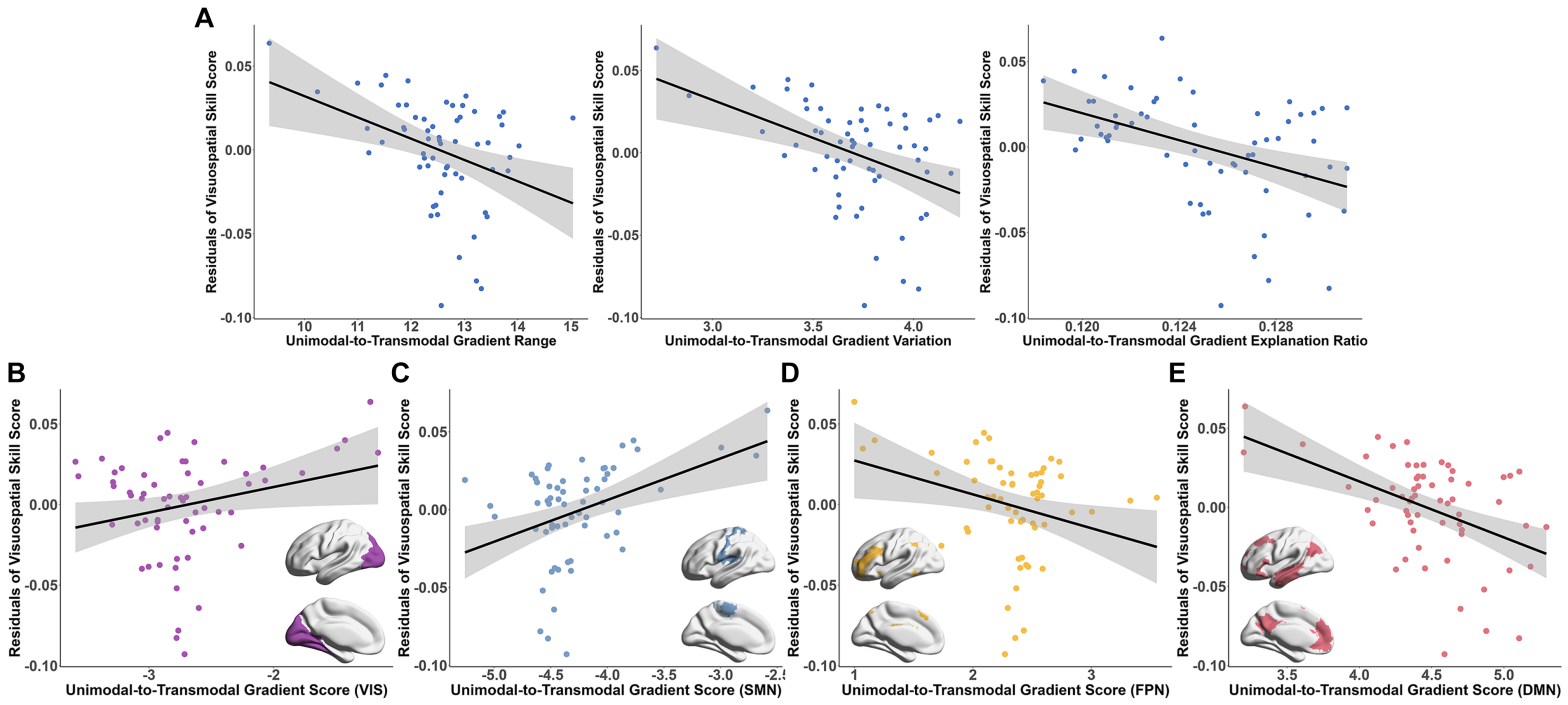
Association between the connectome gradient and the visuospatial skill score of MMSE. **(A)** The association between the principal unimodal-to-transmodal gradient metrics [gradient range (left), variation (middle) and explanation ratio (right)] and the visuospatial skill score residuals. **(B–E)** The association between the gradient scores of VIS **(B)**, SMN **(C)**, FPN **(D)** and DMN **(E)** and the visuospatial skill score residuals. Bonferroni correction *p* < 0.05. VIS, visual network; SMN, somatomotor network; FPN, frontal–parietal network; DMN, default mode network.

Among the MMSE subscale scores, greater visuospatial skill was significantly correlated with the lower degree of principal gradient metrics (gradient range: *t* = −3.942, Bonferroni-corrected *p* = 0.008; variation: *t* = −4.628, Bonferroni-corrected *p* = 7.84 × 10^−4^; explanation ratio: *t* = −5.607, Bonferroni-corrected *p* = 2.26 × 10^−6^, Figure 5A). In the functional subnetworks, the gradient scores of VIS (*t* = 3.68, Bonferroni-corrected *p* = 0.044), SMN (*t* = 5.402, Bonferroni-corrected *p* = 1.12 × 10^−4^), FPN (*t* = −4.224, Bonferroni-corrected *p* = 0.007) and DMN (*t* = −5.082, Bonferroni-corrected *p* = 3.62 × 10^−4^) were significantly associated with the visuospatial skill (Figures 5B–E and Supplementary Table S4). Additionally, there were also relationships between the language ability and the gradient scores of DAN (*t* = −5.73, Bonferroni-corrected *p* = 3.33 × 10^−5^) and LIM (*t* = 4.669, Bonferroni-corrected *p* = 1.58 × 10^−3^; Supplementary Table S4). The association between the connectome gradients and the cognitive scores did not detected at the individual level.

### 3.4 Gender effects on the principal unimodal-to-transmodal gradient

**Figure 6:**
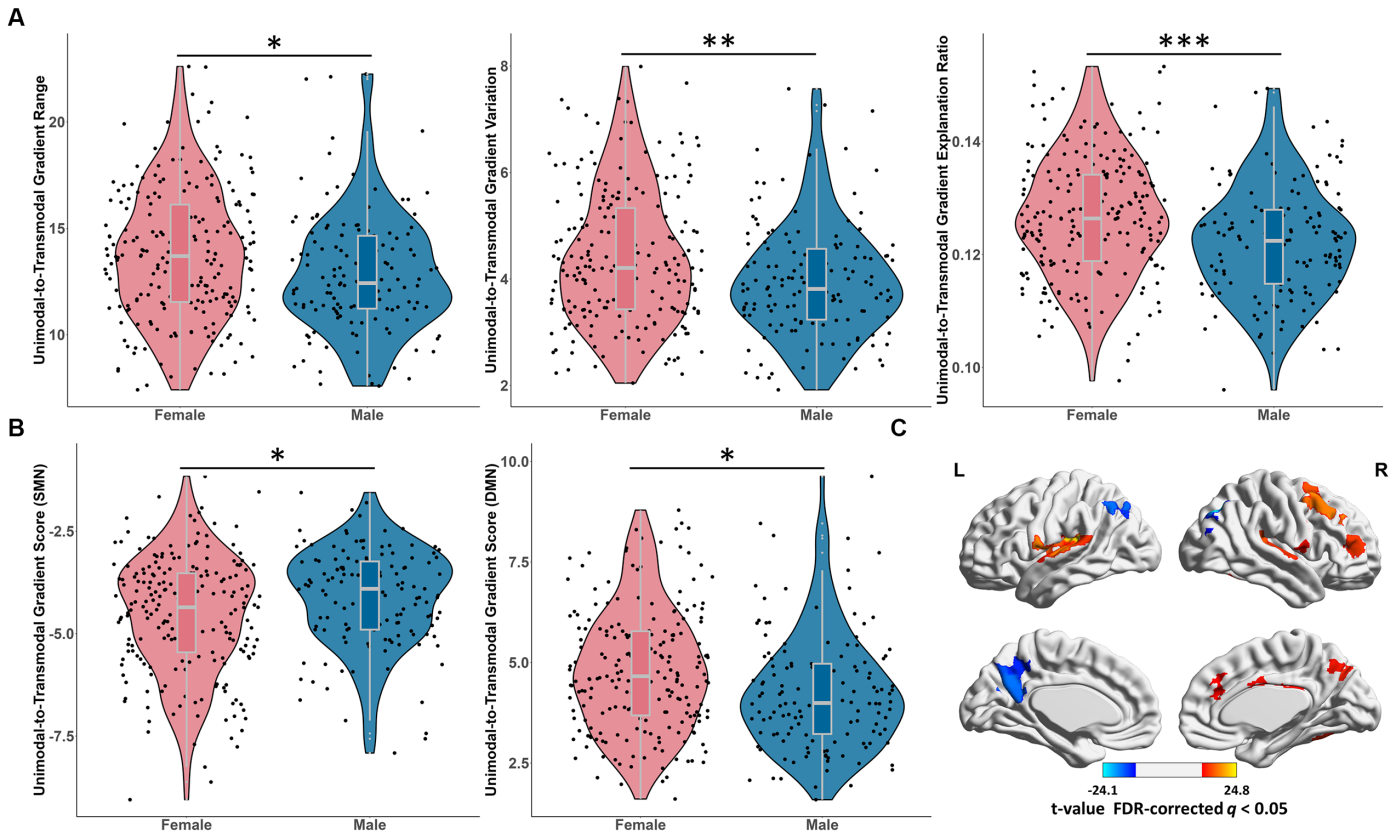
Gender effects on the principal unimodal-to-transmodal gradient. **(A)** The significant gender effects on the principal global gradient metrics, including gradient range, variation and explanation ratio. *p < 0.05; ^**^p < 0.01; ^***^p < 0.001. **(B)** The significant gender differences of the principal gradient scores of SMN and DMN. FDR-corrected *q* < 0.05. ^*^p < 0.05. **(C)** The gender differences of the principal gradient scores in brain regions. The regions showed significantly greater gradient score in males than in females, including STG, MFG, cingulate cortex and Rolandic opercularis; the regions showed significantly greater gradient score in females than in males, including the precuneus, AG, IPL and MOG. Warm color: female < male. Cool color: female > male. FDR-corrected *q* < 0.05. SMN, somatomotor network; DMN, default mode network; STG, superior temporal gyrus; MFG, middle frontal gyrus; AG, angular gyrus; IPL, inferior parietal lobule; MOG, middle occipital gyrus.

We found significant gender effects on the principal gradient range (*t* = 6.26, *p* = 0.013), variation (*t* = 9.51, *p* = 0.0022) and explanation ratio (*t* = 13.53, *p* = 0.0003), with females showing larger values than males (Figure 6A). No age-by-gender interaction effects were observed.

In the principal gradient, the SMN exhibited significantly higher gradient score in male than female (*t* = 8.87, *p*_FDR = 0.028; Figure 6B), while the gradient score of DMN was significantly greater in female than in male (*t* = 10.38, *p*_FDR = 0.025; Figure 6B), and no age-by-gender interaction effects were observed. The gradient score of brain regions showed significantly greater in males than in females were mainly concentrated in STG, middle frontal gyrus (MFG), cingulate cortex and Rolandic opercularis, while the gradient score of the precuneus, angular gyrus (AG), IPL and MOG were significantly greater in females than in males (Figure 6C and Supplementary Table S5). In the secondary gradient, the gradient score of regions (SFG and precuneus) from the attention network were significantly greater in males than in females (Supplementary Table S5).

### 3.5 The potential influences of the cross-age sliding window parameters

Overall, the cross-age sliding window parameters, including the sample size in age windows (Supplementary Figure S4 and Supplementary Table S6), the potential effects of gender (Supplementary Figure S5), the window width (4, 5, 6, 7, 8, 9, 10; Supplementary Figure S6) and step size (1, 2, 3, 4; Supplementary Figure S7) did not affect or alter our main findings. For full results of these validation analysis, see Supplemental materials.

## 4 Discussion

The present study investigated the aging process of the brain’s functional hierarchical organization across the adult lifespan, utilizing cross-age sliding window approach. Our findings demonstrated a significant non-linear aging pattern for the principal unimodal-to-transmodal gradient, contrasted by a linearly decreasing pattern for the secondary visual-to-somatomotor gradient. Furthermore, the changes in the gradient scores of both the somatomotor and frontal–parietal networks were associated with greater working memory and visuospatial ability. The study also revealed that the principal gradient metrics and the gradient scores of SMN and DMN exhibited significant gender differences, which were independent of age. The current findings enrich our comprehension of the aging trajectories of the human brain’s functional hierarchical architecture, which would inform future research on pathological models of atypical aging.

Previous studies on the adult brain have demonstrated the existence and topography of the principal unimodal-to-transmodal and secondary visual-to-somatomotor gradients in the functional connectome (Margulies et al., 2016; Hong et al., 2019; Xia M. et al., 2022). Our study demonstrated that the principal and secondary gradient patterns from early to late adulthood are generally maintained with age. The variances explained by these two gradient components (approximately 24%) were relatively smaller compared to conclusions drawn in other studies (Margulies et al., 2016; Bethlehem et al., 2022; Xia Y. et al., 2022). The lower explanation ratio of the principal gradient may indicate the less differentiated connectivity pattern between the primary and transmodal areas in the studied population, which aligns with a prior research (Xia M. et al., 2022). By investigating the aging trajectories of gradients, we support the findings by Setton et al. (2022) that the gradient pattern may remain stable throughout the lifespan. Moreover, we further demonstrated that the principal gradient may change nonlinearly, differing from secondary gradient, and is associated with cognitive performance. A previous study has examined age effects using cross-sectional resting imaging data in individuals aged 18 to 88 years and detected an increase in dispersion within transmodal communities (DAN, VAN, FPN and DMN), reflecting more diverse functional connectivity profiles within each community (Bethlehem et al., 2020). In contrast to this study, which focused on the ordering of functional networks in a multi-dimensional hierarchical framework, our study specifically uncovered age-related changes at global and local scales in the hierarchical gradient pattern throughout the adult lifespan.

We found that the principal gradient-related metrics change nonlinearly with age, and a notable decline can be observed around the aged of 62. This finding is consistent with, and supported by previous aging studies, which have consistently reported a nonlinear pattern in both brain structural and functional changes, suggesting the age of 60 as a critical point for accelerated aging (Scahill et al., 2003; Ramos et al., 2004; Huang et al., 2018; Zonneveld et al., 2019). Aging studies have also indicated a significant cognitive or behavioral decline occurring around 60 years old (Li et al., 2014; Salthouse, 2019; Nyberg et al., 2020). Such decline includes impairments in processing speed, working memory and long-term memory (Spreng and Turner, 2019), which have greater reliance on executive control supported by the brain function of frontal gyrus. These evidences suggest that normal brain aging predominantly focuses on frontal regions (Buckner, 2004). In our study, we observed significant hierarchical position changes in brain regions along the principal and secondary gradient axis with increasing age, particularly around the medial superior and middle frontal gyrus. This finding aligns with the frontal aging hypothesis, that the frontal cortex is the first to malfunction, leading to a decline in cognitive functions (West, 1996). Furthermore, a recent study has also found a “frontal preservation” pattern characterized by the structural network in elderly individuals who experienced successful cognitive aging (Yang et al., 2022). These findings not only suggest the frontal areas as the key region to deteriorate during the aging process, but also support the impact of brain function deterioration on the hierarchical nature of brain networks.

Compared to younger adults, older adults exhibited higher between-network and lower within-network connectivity in brain connectome (Chan et al., 2014; Geerligs et al., 2015; Stumme et al., 2020). This pattern is generalizable across functional communities (Chan et al., 2014; Damoiseaux, 2017), indicating a reorganization of brain connectivity in older people. In line with previous findings, we observed the narrowing hierarchical distance between unimodal and transmodal areas in the principal and secondary gradient axis, including increasing gradient score of visual and motor areas and decreasing score of frontal, temporal areas and insula. This reflects an increase in between-network connectivity or integration in the older individuals. The increase of between-network connectivity could indicate the age-related alterations in information processing (Chan et al., 2014). For instance, Stumme et al. (2020) found increased FC between brain networks, suggesting a compensation process to maintain the cognitive performance in memory. According to the compensatory theory, older adults tend to recruit more brain regions or initiate a reorganization process in response to high cognitive demand (Cabeza et al., 2018). In particular, older adults may activate more anterior prefrontal regions to compensate for sensory processing deficits in posterior occipital regions when processing cognitive tasks (Li et al., 2015). Therefore, the reorganization process aids in compensating for their higher-order abilities, such as executive and memory functions (Cabeza, 2002; Cabeza et al., 2002; Daselaar et al., 2015; Cabeza et al., 2018), and for impoverished perceptual input, especially in the visual domain (Roberts and Allen, 2016). This compensation mechanism may influence the connectivity pattern between primary and higher-order regions in aging process (Cabeza et al., 2018), potentially giving rise to the relative position changes of these areas.

During the process of aging, the degeneration of working memory is widely regarded as an early sign of cognitive aging, and the associated neuropathological changes are highly vulnerable to both normal and pathological aging processes (Kirova et al., 2015; Zheng et al., 2023). Previous studies found that inferior working memory is linked to the decreased brain modularity, small-worldness, local efficiency and clustering coefficient (Stevens et al., 2012; Langer et al., 2013; Stanley et al., 2015; Finc et al., 2020). In our study, we observed that the reduced segregation between brain networks located at the ends of gradient axis negatively impacts the cognitive performance in the population level. Further analysis indicated that the reduced functional segregation between the SMN and high-order subsystem, i.e., FPN, along the principal gradient axis was related to the degeneration of working memory ability. These findings also correspond well with the “tethering hypothesis,” which contends that the decreased divergence between association and primary cortex may impede the promotion of abstract information integration in the human brain (Buckner and Krienen, 2013; Smallwood et al., 2021). In this situation, the association cortex becomes susceptible to external stimuli interference. Within the current study, the shorter distance between the unimodal and transmodal areas along the principal gradient axis may threaten the performance of higher-order functions. This highlights the crucial role of network segregation in maintaining cognitive function during the normal aging process.

On the contrary, visuospatial skill is an integrative ability that synthesizes some complex cognitive functions such as visual recognition, motor control and working memory (Kravitz et al., 2011; Bradford and Atri, 2014; Tres and Brucki, 2014; Muiños and Ballesteros, 2018) and it supported by the coordinated connectivity of neural circuits in visual, motor, executive control and default mode networks (Kravitz et al., 2011; Garcia-Diaz et al., 2018; Chen et al., 2019). In our study, the principal gradient range served as a reverse representation of the brain integration between functional subnetworks. The decreased relative hierarchical position between VIS, SMN, FPN and DMN on the principal gradient axis resulted in the increase of visuospatial ability, reflecting the involvement of primary and transmodal areas in this combined cognitive process. Furthermore, Han et al. also reported opposite effects for functional connectivity strength in higher- and lower-order brain regions, where increased FC in higher-order regions and decreased FC in lower-order regions are associated with complex cognition in youth adults (Han et al., 2020). This supports our findings that the increase principal gradient range, higher gradient score in transmodal and lower in unimodal regions, are associated with working memory and visuospatial ability. Therefore, an age-related decrease in gradient range may represent reduced network segregation, with proximity in hierarchical positions of higher and lower-order brain regions impacting cognitive ability. Overall, this underscores the value of gradient analysis in the interpreting FC changes, highlighting that different coordination patterns are required in brain networks to support various complex cognitive abilities. Future research should focus on the microstructural changes in brain networks across different functional hierarchies to elucidate the neurobiological mechanisms underlying changes in network hierarchy during aging process.

Our present study also found significant gender-dependent effects on the principal gradient metrics, where the female group showed the wider range, larger variation and higher explanation ratio compared to male participants. This represented the more differentiated connectivity pattern between unimodal and transmodal regions in females and more integrated pattern in males, consistent with previous findings (Satterthwaite et al., 2015; Goldstone et al., 2016; Stumme et al., 2020). Importantly, these gender effects on the principal gradient were independent of aging, indicating that the differences in connectome hierarchical organization persist from early to late adulthood. The identified gender differences in subnetworks’ gradient score were observed in the SMN (male > female) and DMN (female > male) that corroborated the previous works based on the connectome gradient analysis (Liang et al., 2021), machine learning approaches (Zhang et al., 2018; Weis et al., 2020) and brain connectivity (Ritchie et al., 2018). This distinction in brain connectome may partly account for the advantages in specific behaviors and cognitive functions in males or females. The DMN, which is often considered as an important part of the “social brain” (Kennedy and Adolphs, 2012; Mars et al., 2012), may indicate to higher abilities in domains such as social cognition in females, and the SMN may implicate better performance in spatial and motor tasks in males (Gur et al., 2012; Satterthwaite et al., 2015). While our findings align with previous researches on brain function and structure (Satterthwaite et al., 2015; Sang et al., 2021), some studies have reported age-by-gender interactions in brain development and aging (Zuo et al., 2010; Wu et al., 2013). Further investigations using multimodal neuroimaging and behavioral datasets are necessary to gain a better understanding of the gender effects on the brain organization mechanisms across the lifespan.

## 5 Limitations

Several limitations need to be addressed in our study. Firstly, the non-uniform sample size distribution across age windows may affect the final fitting model. We addressed this by randomly selecting five participants from each age window for validation using the same statistical analysis, confirming that our main results are not affected by sample size. However, larger datasets are required to further verify our findings. Secondly, while our aging trajectory analyses of principal gradient-related metrics suggest higher-order regression models may be more suitable, we chose the cubic model for its appropriateness in modeling brain developmental and aging trajectories, as demonstrated in previous studies including evidence from the anatomical structure (Ng et al., 2016; Bethlehem et al., 2022), functional connectome (Zuo et al., 2010; Ng et al., 2016; Bethlehem et al., 2020; Setton et al., 2022) and topological properties (Cao et al., 2014; Chan et al., 2014). Moreover, the cubic model also showed statistical optimality in the bootstrap and validation analyses, particularly for modeling changes in gradient range, a key metric for functional hierarchical architecture. Thirdly, we currently used MMSE subscales to determine cognitive relevance of brain functional hierarchy. Subsequent research may consider assessing different domains of cognitive abilities and enhancing the specificity and generality of findings in both context of normal and pathological aging process. Lastly, our results are based on the population cohort, and the discussion on aging process and its cognitive implications are restricted to the population level, rather than representing individualized aging processes. Future longitudinal studies will be needed to confirm whether connectome gradients are sensitive enough to detect changes in functional reorganization during aging in healthy or diseased individuals.

## 6 Conclusion

To summarize, we delineated the aging-related changes in the principal unimodal-to-transmdodal and secondary visual-to-somatomotor functional connectome gradients throughout the adult lifespan. Additionally, we demonstrated the independent effect of gender on network hierarchical organization. The principal gradient captured the topographical property of functional networks and was associated with the decline of working memory and visuospatial abilities. These findings provide evidence for further investigations into brain function and its biological basis, offering important implications for developing precise strategies to maintain cognitive ability throughout the lifespan in both health and disease.

## Data availability statement

The original contributions presented in the study are included in the article/Supplementary material, further inquiries can be directed to the corresponding authors.

## Ethics statement

The study obtained ethical approval from the Institutional Review Board of Taipei Veterans General Hospital and all participants provided written informed consent.

## Author contributions

XW: Conceptualization, Data curation, Formal analysis, Investigation, Methodology, Project administration, Software, Validation, Visualization, Writing – original draft, Writing – review & editing. C-CH: Conceptualization, Funding acquisition, Investigation, Methodology, Project administration, Resources, Writing – review & editing. S-JT: Data curation, Funding acquisition, Methodology, Writing – review & editing. C-PL: Data curation, Funding acquisition, Methodology, Writing – review & editing. QC: Conceptualization, Project administration, Supervision, Writing – review & editing.

## Footnotes

^1^ https://brainspace.readthedocs.io/en/latest/index.html

^2^ https://sites.google.com/site/bctnet/

^3^ https://www.mathworks.com/matlabcentral/fileexchange/27014-mancovan
