# Supplementary material for: The aging trajectories of brain functional hierarchy and its impact on cognition across the adult lifespan
Source: Front Aging Neurosci. 2024 Jan 19;16:1331574. doi: 10.3389/fnagi.2024.1331574 (PMC10837851; doi:10.3389/fnagi.2024.1331574)
Supplement: Supplementary file 1 [file Data_Sheet_1.docx]

Supplemental material

# Materials and methods

## Imaging data preprocessing

MRI images preprocessing was performed using fMRIPrep 20.2.1 (<https://fmriprep.org/en/stable/>) (Esteban et al., 2019). Each T1w image was corrected for intensity non-uniformity (INU) with *N4BiasFieldCorrection*, distributed with ANTs; skull-stripped with a Nipype implementation of the *antsBrainExtraction.sh* workflow (ANTs), using *OASIS30ANTs* as target template. Brain tissue segmentation of cerebrospinal fluid (CSF), white-matter (WM) and gray-matter (GM) was performed on the brain-extracted T1w using *fast* (FSL). Volume-based spatial normalization to the Montreal Neurological Institute (MNI) standard space (MNI152NLin2009cAsym) was performed through nonlinear registration with *antsRegistration* (ANTs).

The overall resting-state images preprocessing included the following steps: removal of the first 10 volumes from the whole series; slice-timing correction using *3dTshift* from AFNI; head motion correction using *mcflirt* (FSL); co-registration to the T1w images using *flirt* (FSL) with the boundary-based registration cost-function; physiological noise regressors extraction using component-based noise correction (CompCor (Behzadi et al., 2007)); and estimation of several confounding parameters and time-series (including frame-wise displacement (FD), DVARS, global signals, mean WM and CSF signals). The spatial smoothing was performed with a Gaussian-smoothing kernel of 6mm full-width at half-maximum (FWHM) using *3dmerge* in AFNI. Confounds included global signal, the 12 head-motion parameters (6 basic motion parameters + 6 temporal derivatives), signals from 6 aCompCor components, 6 discrete cosine-basis regressors and 10 dummy scans (non_steady_state_outlier) were regressed from the preprocessed resting-state data for each individual using *3dTproject* in AFNI. Finally, we excluded 5 participants with brain structural abnormalities, and 26 participants with mean FD larger than 0.3mm, or the max head motion above 1.5mm or 1.5 degrees, and or more than 10% of the frames FD above 0.5mm, remaining 356 participants.

## Connectome gradient analysis

We constructed a region-wise FC matrix for each participant based on the Schaefer’s cortical parcellation map of 400 regions (Schaefer et al., 2017) by computing the Pearson correlation coefficients (converted to Fisher’s Z-values) between the averaged time series of each pair of brain regions. The functional connectome gradients (Margulies et al., 2016) were estimated using the BrainSpace toolbox (<https://brainspace.readthedocs.io/en/latest/index.html>)(Vos de Wael et al., 2020). The FC profile vector of each brain region was thresholded by retaining the top 10% strongest connections and the remaining connections were set to zero, as was done previously (Margulies et al., 2016). We then calculated the cosine similarity matrix that captures similarity in connectivity patterns between each pair of regions. The diffusion embedding mapping algorithm with a manifold learning parameter of *α* = 0.5 (Hong et al., 2019; Margulies et al., 2016) was then applied to the similarity matrix to identify multiple low-dimensional gradient components. To ensure the comparability of the gradient pattern among participants, we used the Procrustes rotation alignment approach to align each individual’s original gradient distribution pattern to a group-level gradient template that based on the overall healthy adults. For each connectome gradient, a gradient score was assigned to each brain region, which represents the relative hierarchical position of brain regions along the gradient axes.

To quantify the global connectome gradient pattern, we calculated the global gradient metrics (post-alignment), including gradient explanation ratio, range and variation for all participants. The gradient explanation ratio, defined as the eigenvalue of the given gradient divided by the sum of all eigenvalues, represents the percentage of connectivity variance accounted for by that gradient. The gradient range indicates the difference of gradient scores in the encoded connectivity pattern between the regions localized at the gradient ends. The gradient variation, i.e. the variance of the given gradient, reflects the heterogeneity in the connectivity structure across regions. Six participants were removed based on the general criteria of mean ± 3 standard deviations from the distributions of gradient ranges and variations in all participants. Finally, this study included 350 participants (age = 44.74 ± 15.91 years, female = 213) for further analyses.

## Statistical analysis

## The potential influences of the cross-age sliding window parameters

To ascertain the robustness of the main results and examine the potential influences of the cross-age sliding window parameters, we performed the following analyses. First, to exclude the effects of the sample size in age windows on the final model selection, we selected 5 participants randomly in the first 50 windows (the rest of 12 windows contains less than or equal to 5 participants) and fitted the linear and polynomial models. Second, we examined the potential effects of gender by repeating the cross-age sliding window analysis separately for female and male (213 female and 137 male participants). Consistent with the main analysis, we defined the age window spanning 5 years and taking a step size of 1 years. We fitted the cubic model and linear model respectively for principal and secondary gradient metrics, and focused the effects of age, gender and the age-by-gender interaction. Finally, we investigated the effects of the window width and step size of the sliding window on aging trajectory. Based on the main results, we systematically varied the parameters of sliding window by “fixing the window width, changing the step size” (window width = 5 years, step size = 1, 2, 3, 4 years) and “fixing the step size, changing the window width” (window width = 4, 6, 7, 8, 9, 10 years, step size = 1years) and repeated the cross-age sliding window analysis.

# Results

## The aging process of functional connectome gradients

At the individual level, the principal gradient explanation ratio showed significant increases with age (t = 4.774, *p* = 2.68 × 10^-6^), while the gradient range (t = 1.096, *p* = 0.274) and variation (t = 1.288, *p* = 0.199) showed none (Figure S1 A). In the secondary gradient, the global range (t = -4.199, *p* = 3.41 × 10^-5^) and variation (t = -3.685, *p* = 2.65 ×10^-4^) showed significant decrease with age, while the explanation ratio was not related to age (t = -1.479, *p* = 0.14. Figure S1 B). The gradient score of DAN in the principal gradient decreased with age (t = -3.53, *p*-FDR = 0.0086. Figure S2 A). In the secondary gradient, the gradient score of VIS increased with age (t = 4.215, *p*-FDR = 5.8 × 10^-4^), and VAN decreased with age (t = -3.91, *p*-FDR = 0.0001, Figure S2 B). At the region level, the principal gradient score of regions from DAN and DMN were negatively associated with age after FDR correction (Figure S2 C). In the secondary gradient, the gradient score of regions from VIS were positively correlated with age, and regions in SMN, VAN and DMN were negatively correlated with age after FDR correction (Figure S2 C).

## The potential influences of the cross-age sliding window parameters

To eliminate the influence of the sample size in age windows on the model selection, we randomly selected 5 participants in each window to identify the aging process of global gradient metrics. The results showed that, consistent with our main results, the age effect of principal gradient metrics was more suitable for fitting models with cubic or higher degree, and the secondary gradient metrics still maintained the linear process (Figure S4 and Table S6).

To investigated the gender-dependent aging process of the connectome gradient, we repeated the cross-age sliding window analysis separately for each gender and estimated the effects of gender and age-by-gender interaction. In the principal gradient, significant gender effects were observed on the gradient variation (t = 2.74, *p* = 0.0072) and explanation ratio (t = 5.292, *p* = 6.15 × 10^-7^), and there was also a significant age-by-gender interaction in explanation ratio (t = 2.584, *p* = 0.011, Figure S5). In the secondary gradient, the gradient explanation ratio showed the significant gender effect (t = 2.166, *p* = 0.032) and age-by-gender interaction (t = 2.133, *p* = 0.035, Figure S5). Although we found some gender and age-by-gender interaction effects on the principal and secondary gradient metrics, the aging trajectories for male and female in the cross-age sliding window was convergent, similar to our main results.

Finally, to evaluated robustness of our main findings, we investigated the impact of sliding window parameters by systematically varying the window width (4, 6, 7, 8, 9, 10, Figure S6) and step size (1, 2, 3, 4, Figure S7) respectively. Based on the model criterions (AIC, BIC, RMSE and R-squared), results were qualitatively consistent with the main findings, showing nonlinear aging trajectories for the principal gradient metrics and linear trajectories for the secondary gradient metrics.


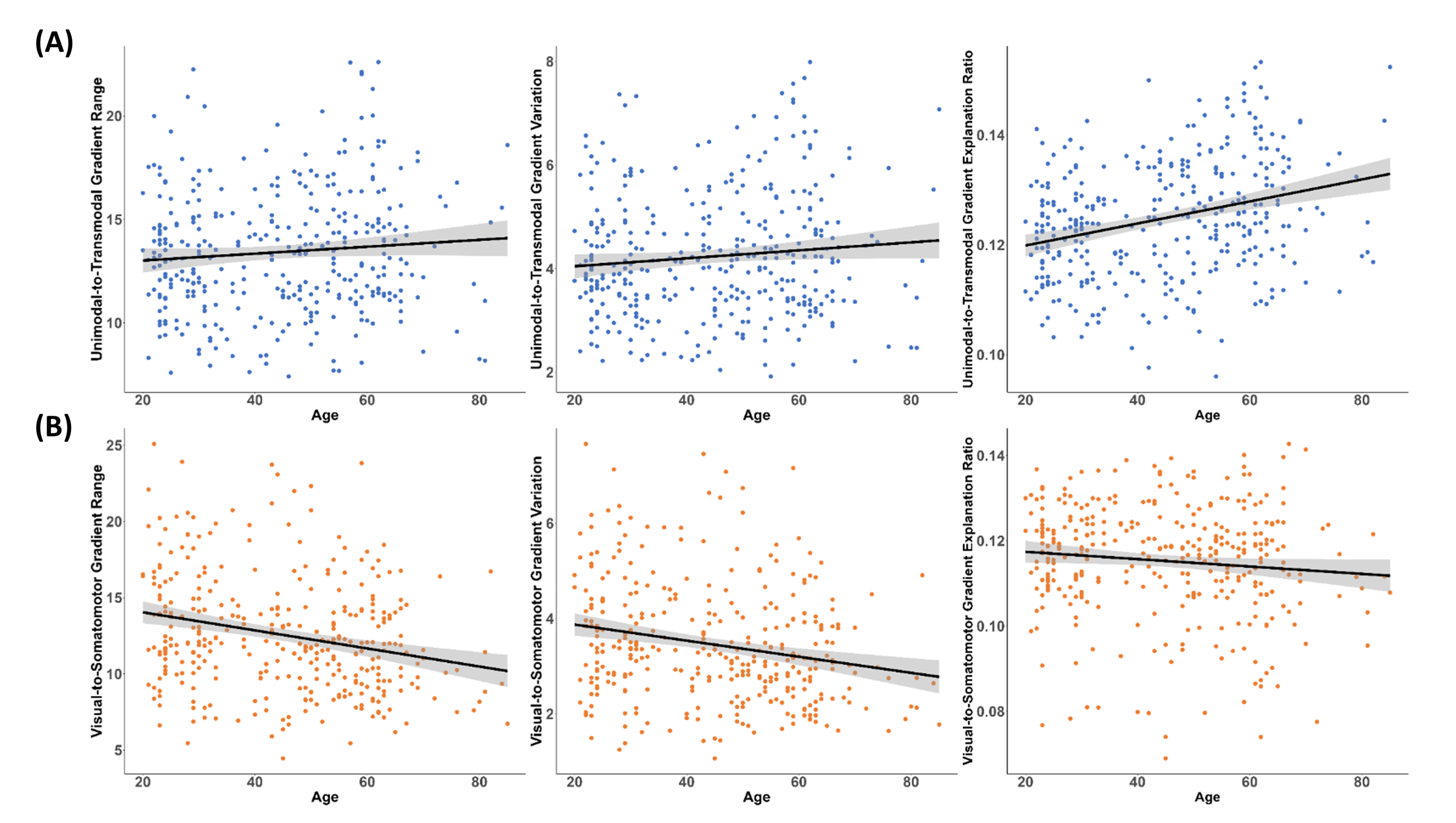


**Supplementary Figure 1.** The aging trajectories of functional connectome gradient global metrics at the individual level. (A) The association between age and the principal unimodal-to-transmodal gradient metrics, including gradient range (t = 1.096, *p* = 0.274), variation (t = 1.288, *p* = 0.199) and explanation ratio (t = 4.774, *p* = 2.68 × 10^-6^). (B) The association between age and the secondary visual-to-somatomotor gradient metrics, including gradient range (t = -4.199, *p* = 3.415 × 10^-5^), variation (t = -3.685, *p* = 2.65 × 10-4) and explanation ratio (t = -1.479, *p* = 0.14). Blue dots: the principal gradient metrics. Orange dots: the secondary gradient metrics.


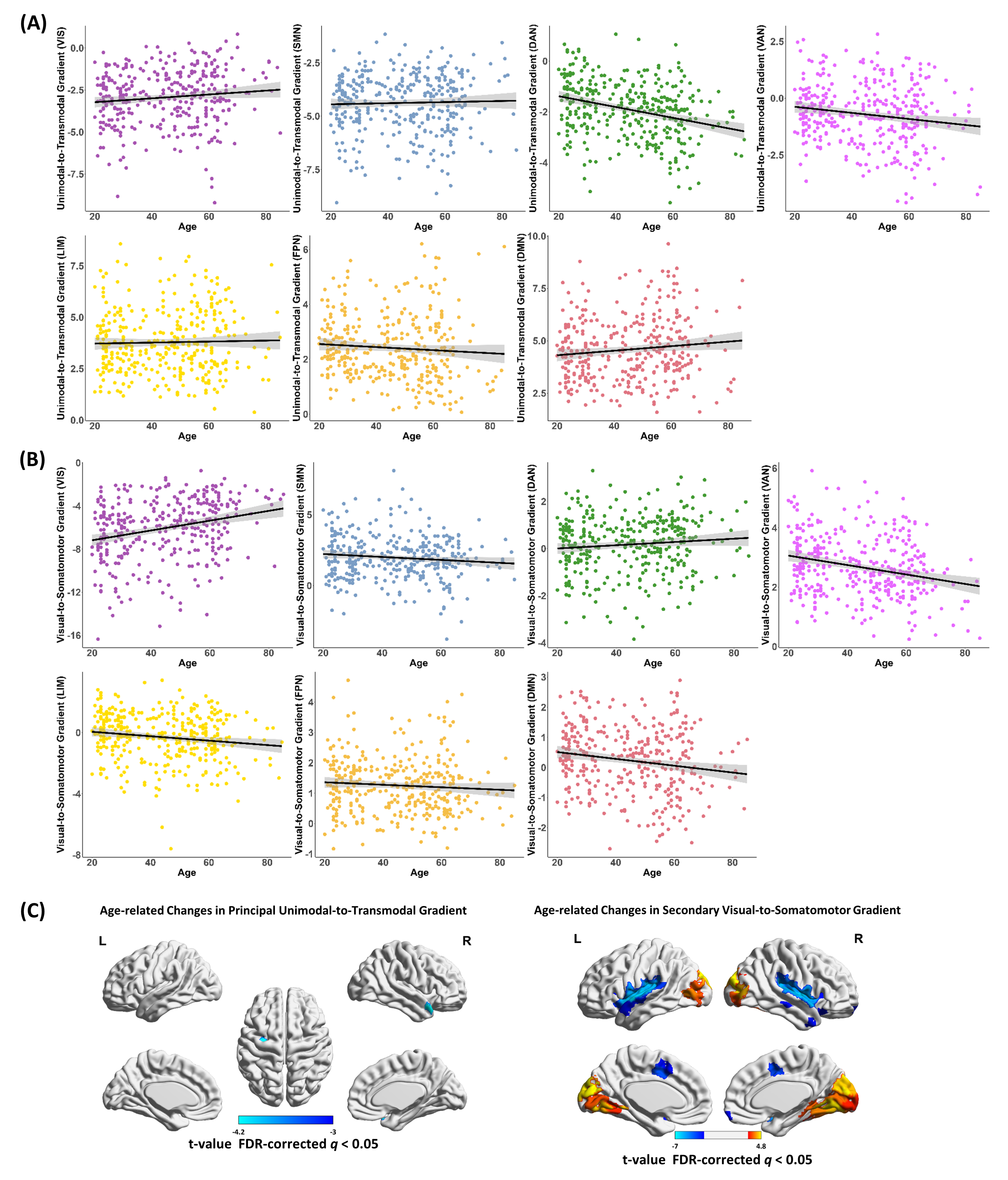


**Supplementary Figure 2.** The age-related changes in functional connectome gradients at the individual level. (A) The association between age and principal gradient score in functional subnetworks. VIS: t = 1.53, *p*-FDR = 0.768; SMN: t = 0.272, *p*-FDR = 1; DAN: t = -3.53, *p*-FDR = 0.0086; VAN: t = -2.196, *p*-FDR = 0.261; LIM: t = -0.087, *p*-FDR = 1; FPN: t = -0.636, *p*-FDR = 1; DMN: t = 1.339, *p*-FDR = 0.823. FDR-corrected *q* < 0.05. (B) The association between age and secondary gradient score in functional subnetworks. VIS: t = 4.215, *p*-FDR = 5.8 × 10^-4^; SMN: t = -2.115, *p*-FDR = 0.128; DAN: t = 1.393, *p*-FDR = 0.498; VAN: t = -3.906, *p*-FDR = 0.001; LIM: t = -2.273, *p*-FDR = 0.107; FPN: t = -0.879, *p*-FDR = 0.986; DMN: t = -2.307, *p*-FDR = 0.131. FDR-corrected *q* < 0.05. (C) Age-related changes in the principal (left) and secondary (right) gradient score in brain regions. FDR-corrected *q* < 0.05. Abbreviations: VIS, visual network; SMN, somatomotor network; DAN, dorsal attention network; VAN, ventral attention network; LIM, limbic network; FPN, frontal-parietal network; DMN, default mode network.


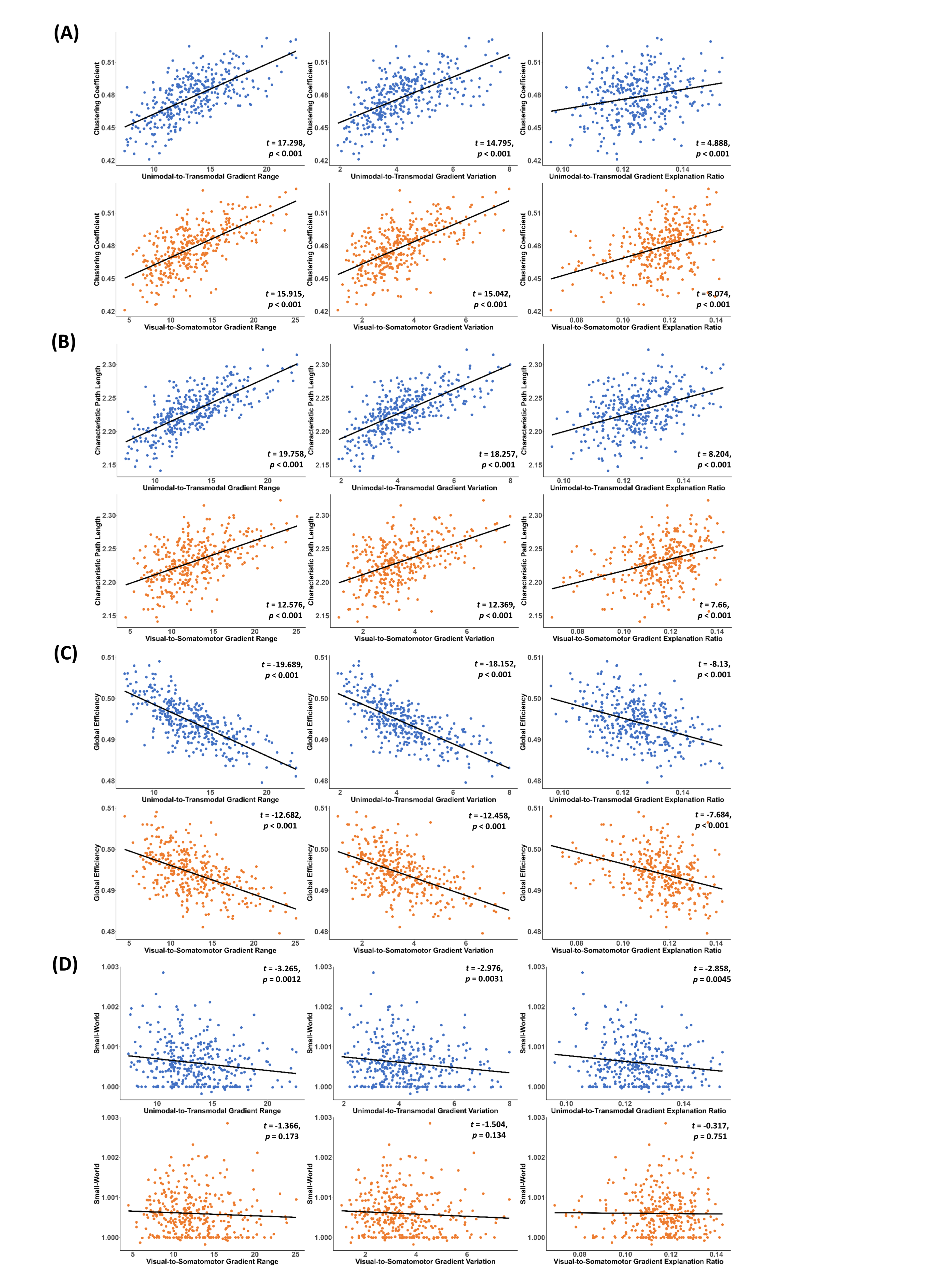


**Supplementary Figure 3.** Association between the global gradient metrics and functional network topographical properties. The association between the global gradient metrics (gradient range, variation and explanation ratio) and clustering coefficient (A), characteristic path length (B), global efficiency (C) and small-worldness property (D). Blue dots: the principal gradient. Orange dots: the secondary gradient.


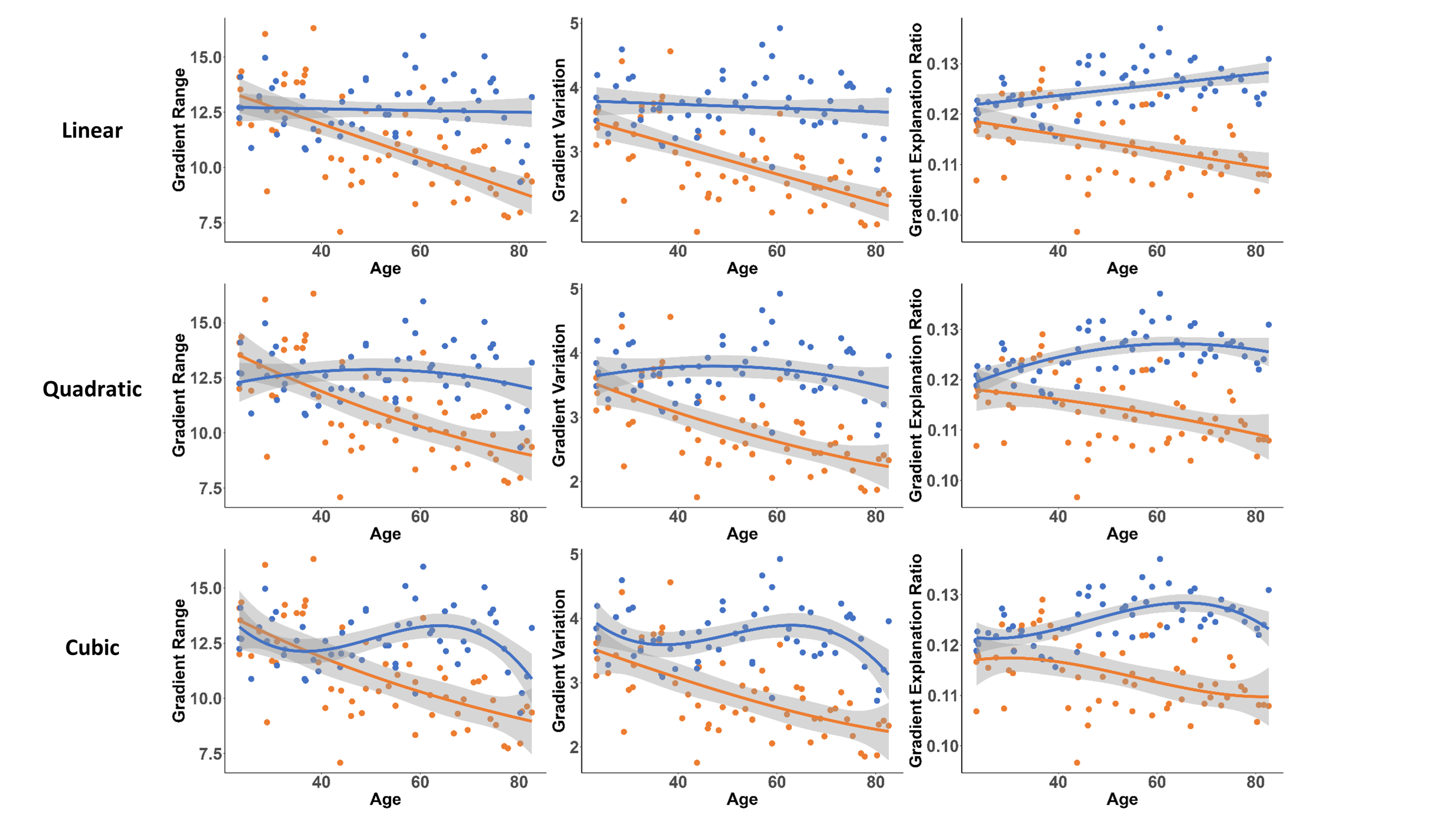


**Supplementary Figure 4.** The aging trajectories of global gradient metrics (gradient range, variation and explanation ratio) by randomly selecting 5 participants in cross-age sliding windows, fitted using linear (top), quadratic (middle) and cubic (bottom) regression models with education level and mean FD parameters as covariates. Blue dots and lines: the principal gradient. Orange dots and lines: the secondary gradient.


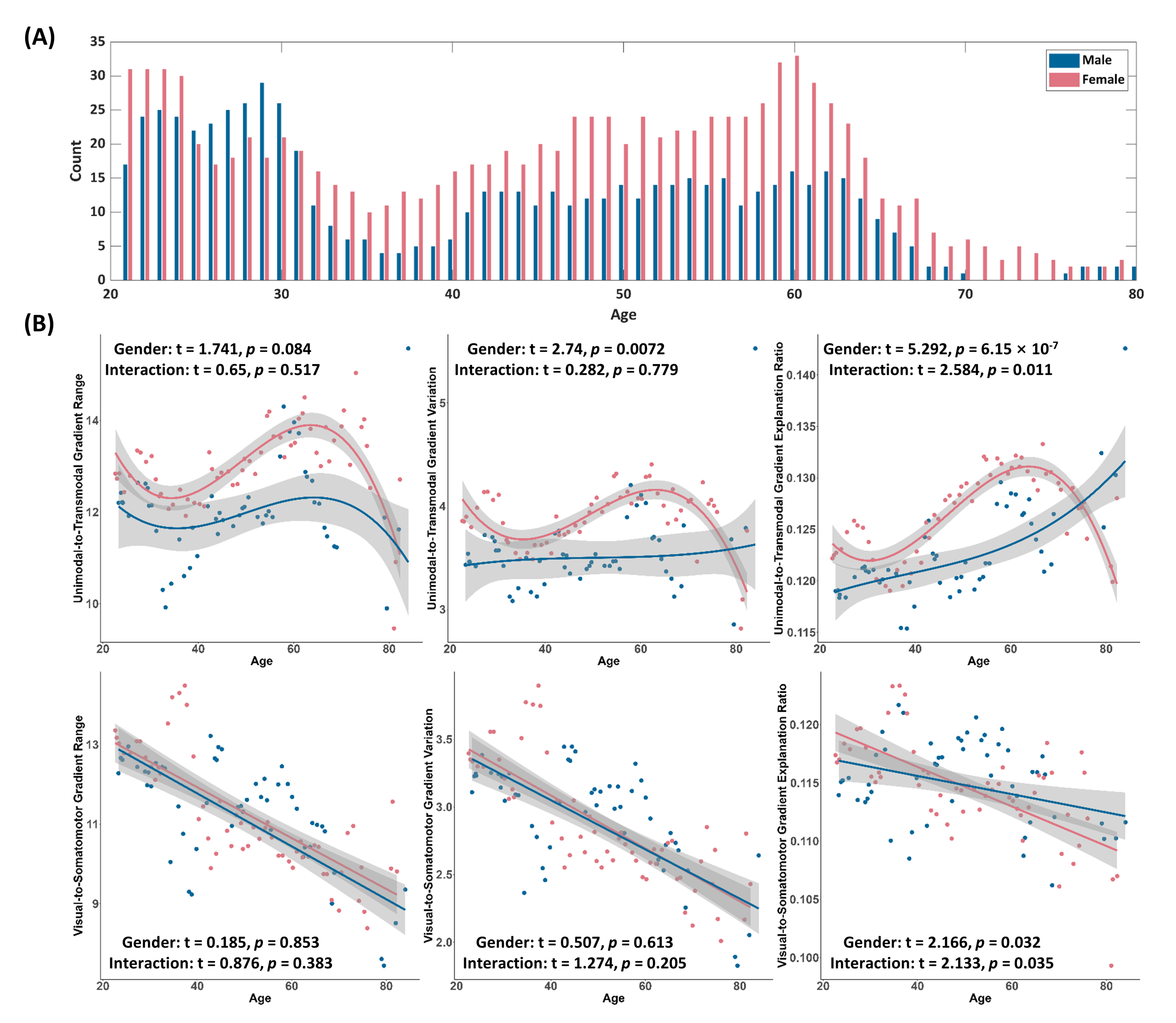


**Supplementary Figure 5.** The gender effect on the aging trajectories of global gradient metrics. (A) The number of female and male participants in each age window. (B) The aging trajectories of principal (top) and secondary gradient metrics (bottom) for female and male fitted using cubic and linear regression models. Pink dots and lines: female; Blue dots and lines: male.


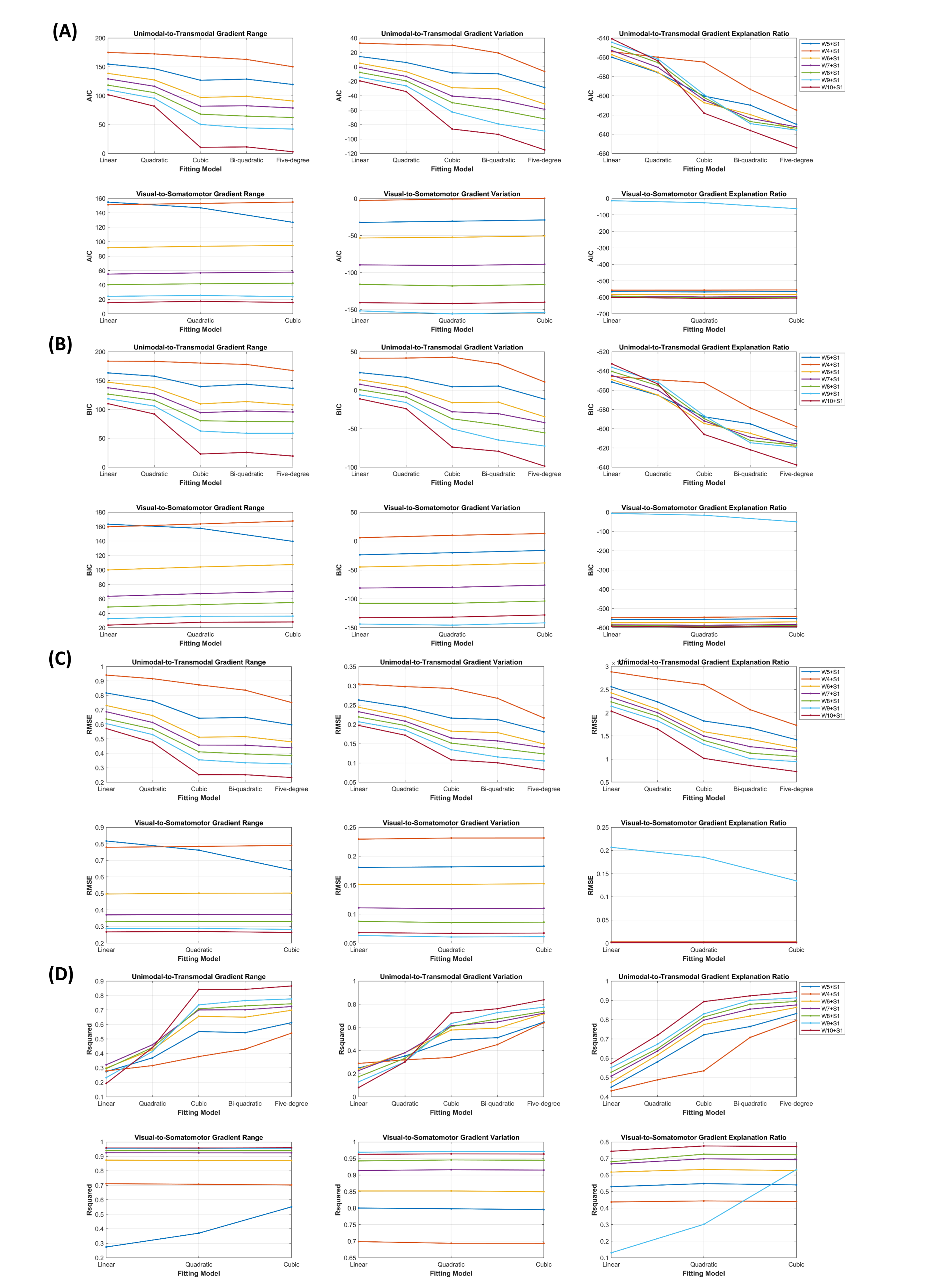


**Supplementary Figure 6.** The goodness-of-fit metrics for generalized linear regression models. The line chart of model criterions, including AIC (A), BIC (B), RMSE (C) and R-squared (D), in the association between age and the principal (top) and secondary (bottom) gradient metrics (gradient range, variation and explanation ratio) when varying the window width (4, 6, 7, 8, 9, 10) in the cross-age sliding window analysis. Abbreviations: RMSE, root mean squared error; AIC, Akaike information criterion; BIC, Bayesian Information Criterions; W, window width; S: step size.


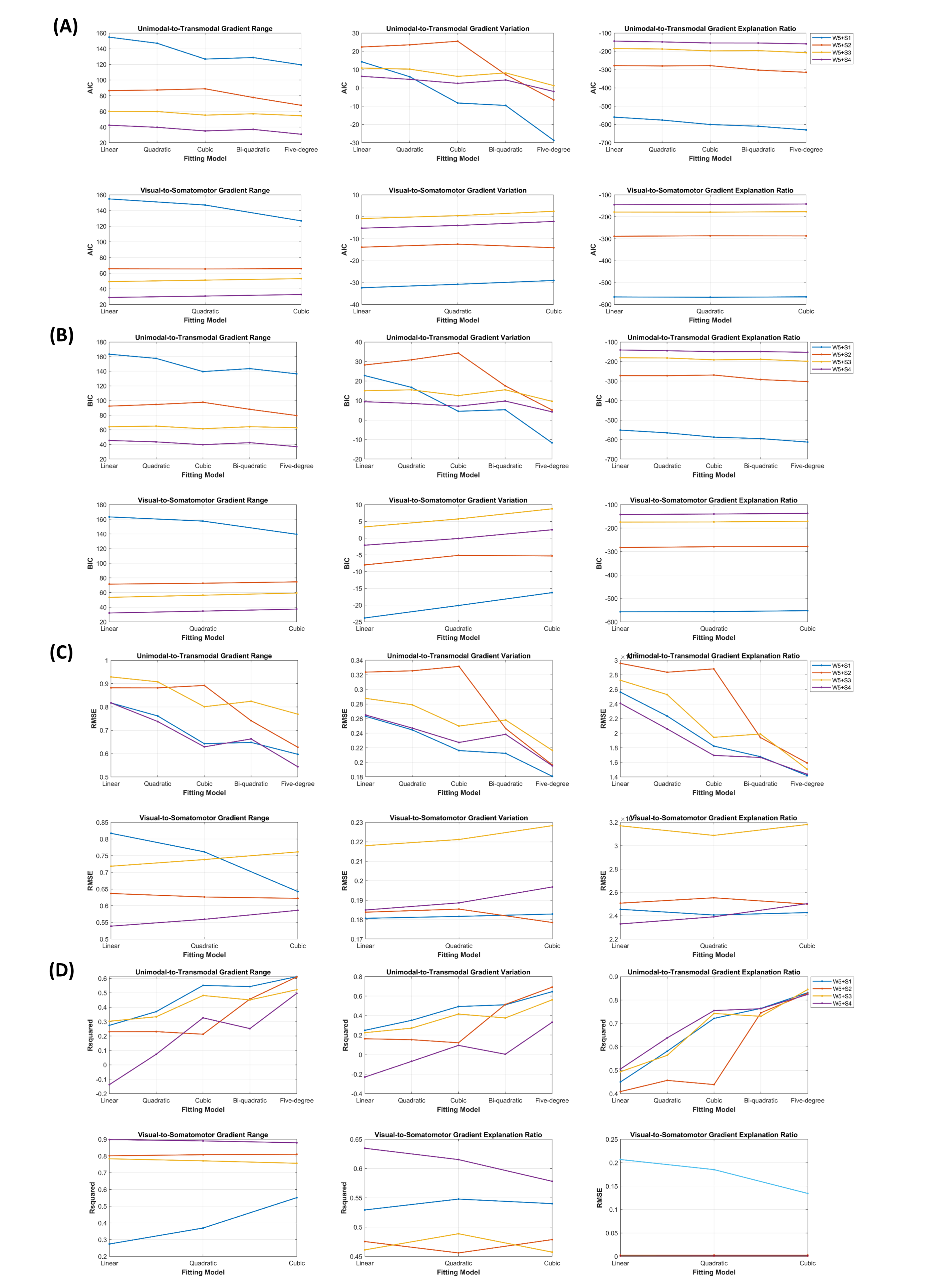


**Supplementary Figure 7.** The goodness-of-fit metrics for generalized linear regression models. The line chart of model criterions, including AIC (A), BIC (B), RMSE (C) and R-squared (D), in the association between age and the principal (top) and secondary (bottom) gradient metrics (gradient range, variation and explanation ratio) when varying the step size (1, 2, 3, 4) in the cross-age window analysis. Abbreviations: RMSE, root mean squared error; AIC, Akaike information criterion; BIC, Bayesian Information Criterions; W, window width; S: step size.

**References**

Behzadi, Y., Restom, K., Liau, J., & Liu, T. T. (2007). A component based noise correction method (CompCor) for BOLD and perfusion based fMRI. *Neuroimage*, *37*(1), 90-101. <https://doi.org/10.1016/j.neuroimage.2007.04.042>

Esteban, O., Markiewicz, C. J., Blair, R. W., Moodie, C. A., Isik, A. I., Erramuzpe, A., Kent, J. D., Goncalves, M., DuPre, E., Snyder, M., Oya, H., Ghosh, S. S., Wright, J., Durnez, J., Poldrack, R. A., & Gorgolewski, K. J. (2019). fMRIPrep: a robust preprocessing pipeline for functional MRI. *Nat Methods*, *16*(1), 111-116. <https://doi.org/10.1038/s41592-018-0235-4>

Hong, S. J., Vos de Wael, R., Bethlehem, R. A. I., Lariviere, S., Paquola, C., Valk, S. L., Milham, M. P., Di Martino, A., Margulies, D. S., Smallwood, J., & Bernhardt, B. C. (2019). Atypical functional connectome hierarchy in autism. *Nat Commun*, *10*(1), 1022. <https://doi.org/10.1038/s41467-019-08944-1>

Margulies, D. S., Ghosh, S. S., Goulas, A., Falkiewicz, M., Huntenburg, J. M., Langs, G., Bezgin, G., Eickhoff, S. B., Castellanos, F. X., Petrides, M., Jefferies, E., & Smallwood, J. (2016). Situating the default-mode network along a principal gradient of macroscale cortical organization. *Proc Natl Acad Sci U S A*, *113*(44), 12574-12579. <https://doi.org/10.1073/pnas.1608282113>

Schaefer, A., Kong, R., Gordon, E. M., Laumann, T. O., Zuo, X.-N., Holmes, A. J., Eickhoff, S. B., & Yeo, B. T. T. (2017). Local-Global Parcellation of the Human Cerebral Cortex from Intrinsic Functional Connectivity MRI. *Cerebral Cortex*, *28*(9), 3095-3114. <https://doi.org/10.1093/cercor/bhx179>

Vos de Wael, R., Benkarim, O., Paquola, C., Lariviere, S., Royer, J., Tavakol, S., Xu, T., Hong, S. J., Langs, G., Valk, S., Misic, B., Milham, M., Margulies, D., Smallwood, J., & Bernhardt, B. C. (2020). BrainSpace: a toolbox for the analysis of macroscale gradients in neuroimaging and connectomics datasets. *Commun Biol*, *3*(1), 103. <https://doi.org/10.1038/s42003-020-0794-7>
